# Toward computational modelling on immune system function

**DOI:** 10.1186/s12859-019-3239-x

**Published:** 2019-12-10

**Authors:** Francesco Pappalardo, Marzio Pennisi, Pedro A. Reche, Giulia Russo

**Affiliations:** 1 Department of Drug Science, University of Catania, V.le A. Doria 6, Catania, 95125 Italy; 2 Department of Mathematics and Computer Science, University of Catania, V.le A. Doria 6, Catania, 95125 Italy; 3 Universidad Complutense de Madrid, Facultad de Medicina,Departamento de Immunología, Plaza Ramón y Cajal, Madrid, 28040 Spain

## Abstract

The 2nd Computational Methods for the Immune System function Workshop has been held in Madrid in conjunction with the IEEE International Conference on Bioinformatics and Biomedicine (BIBM 2018) in Madrid, Spain, from December 3 to 6, 2018. The workshop has been obtained 100% more submissions in respect to the first edition, highlighting a growing interest for the treated topics. The best papers (9) have been selected for extension in this special issue, with themes about immune system and disease simulation, computer-aided design of novel candidate vaccines, methods for the analysis of immune system involved diseases based on statistical methods, meta-heuristics and game theory, and modelling strategies for improving the simulation of the immune system dynamics.

## Introduction

The constant and rapid increasing of computing power has favoured the diffusion of computational methods into immunology, giving the birth to computational immunology. Computational immunology, known also as immunological bioinformatics or immunoinformatics, is a modern research area that embraces data-driven computational and mathematical approaches able to model and describe the dynamics of cellular and molecular entities of the immune system, its disorders, and infections. Nowadays, immunoinformatics is dedicated to methods and tool developments for the analyses of omic-type data in immunology and knowledge inference using simulation, statistical inference, and machine learning algorithms. These fields are complementary key drivers of data-driven basic and translational immunology research for the benefit of human and animal health.

The idea of a workshop to focus on the application of computer methods and computational models, at any level of description (e.g., microscopic/intracellular, mesoscopic/intercellular, macroscopic/tissue or organs), for the modeling of the Immune system function, along with their application in understanding the pathogenesis of specific diseases (e.g., infectious diseases, cancers, hypersensitivities, autoimmune disorders) has been in our minds for a long time and has been then realized, for the first time, in 2018.

Then, the second edition of Computational Methods for the Immune System Function (CMISF) has been held in Madrid in conjunction with the IEEE International Conference on Bioinformatics and Biomedicine (BIBM 2018) on December 3, and it has been able to obtain 100% more submissions than the first editon, moving to a full day workshop. The most promising contributions that were in line with the topics of interest of the workshop have been selected and invited to submit an extended version of their work, for a total of 8 submissions. Author contributions were from Europe, Asia, Oceania, North and South America, for an almost worldwide coverage.

## Topics covered

The main topics of interest regarded the simulation of pathologies that involve the Immune System as solution or cause of the disease, but also contributions about the optimization of simulation methodologies, about the definition of novel vaccine candidates using computational methods, methods based on statistical techniques, meta-heuristics, or evolutionary game theory for the analysis of diseases and IS behavior have been presented.

A prominent topic refers to one of the most important auto-immune diseases, Multiple Sclerosis (MS). In their work, Pernice et al. [1] present a new methodology for studying Relapsing Remitting Multiple Sclerosis (RRMS), implemented using GreatSPN, a state-of-the-art open-source suite for modelling and analyzing complex systems through the Petri Net (PN) formalism. Thanks to the use of Colored Petri Nets for the developing of the model and Latin Hypercube Sampling with Partial Rank Correlation Coefficients to calibrate model parameters on real behaviours of healthy and MS subjects, the methodology allowed to analyze both the effect of the daclizumab administration and the behavior of RRMS in pregnant women.

A study about computational modeling of myocarditis was instead presented Reis et al. [2]. In their work the authors show a new hydro-mechanical model for inflammatory edema that combines a nonlinear system of partial differential equations (PDE) based on porous media approach with a model of the immune response involved in myocarditis. To validate the approach a T2 parametric mapping obtained by Magnetic Resonance (MR) imaging was used to identify the region of edema in a patient diagnosed with unspecific myocarditis. The use of the computational approach allowed to reproduce the edema formation, allowing to correlate spatiotemporal dynamics of representative cells of the immune systems, such as leucocytes and the pathogen, with fluid accumulation and cardiac tissue deformation.

Pennisi et. al. [3] presented an extension of the Universal Immune System Simulator (UISS) for modeling of Tuberculosis. The model demonstrated capable of simulating the main features and dynamics of the mycobacterium tuberculosis. Furthermore, the artificial immunity elicited by the use of the RUTI vaccine, a polyantigenic liposomal therapeutic vaccine made of fragments of mycobacterium tuberculosis cells (FCMtb), was also analysed. The application of these computational modeling strategies like the ones presented here helpfully contribute to open the door for a prompt integration of in silico methods within the pipeline of clinical trials, supporting and guiding the testing of novel treatments.

An exploratory analysis using a correlation-based network on five different datasets obtained from 11 obese with metabolic syndrome (MetS) and 12 healthy weight men has been performed by Chen et al. [4]. Their findings show a possible high involvement of immune cells in obese with MetS individuals, suggesting a more complex interaction of inflammatory markers in obesity, with high connectivity of immune cells in the obese with MetS network compared to the healthy network. It is worth to note that, compared to a t-test, the network approach offered more meaningful results when comparing obese with MetS and healthy weight individuals.

In their work, Zhang et al. [5] show how the use of Genetic algorithms (GAs) can improve the research of the best combination of correlated variables in Principal components analysis (PCA). PCA is often used to find characteristic patterns associated with certain diseases by reducing variable numbers before a predictive model is built, particularly when some variables are correlated. Usually the first two or three components from PCA are used to determine whether individuals can be clustered into two classification groups. The developing of a prediction model that combines PCA and a genetic algorithm (GA) for identifying sets of bacterial species associated with obesity and metabolic syndrome (Mets), offered a new way to build prediction models that may improve the prediction accuracy, overcoming the limitations of PCA for data analysis.

A novel multiantigenic epitope vaccine ensemble against the Human Cytomegalovirus was instead presented by Quinzo et al. [6]. Such candidate vaccine, that should elicit T and B cell responses in the entire population, has been totally designed with the help of a computer-assisted approach that feeds on previously identified epitopes readily available in specialized databases. The keystone of this approach is to select conserved epitopes that are likely to induce protective immune responses.

Presbitero et al. [7] applied evolutionary game theory to study the balance between apoptosis and necrosis of Neutrophils during inflammation. Thanks to their approach the authors were able to formulate a game that predicts the percentage of necrosis and apoptosis when exposed to various levels of insults, identifying the driving mechanisms that lead to the delicate balance between apoptosis and necrosis in neutrophils’ cell death. Moreover, this simple model allowed the verify that global cost of remaining inflammation triggering moieties is the driving mechanism that reproduces the percentage of necrosis and apoptosis observed in data.

Finally Chimeh et al. [8] focused on the improvement of Agent Based Modeling (ABM) techniques for simulating massive multi-agent systems (i.e. simulations containing a large number of agents/entities), as these require major computational effort, which is only achievable through the use of parallel computing approaches, to simulate large scale biological systems. In particular different approaches to parallelising the key component of biological and immune system models within an ABM model, i.e. pairwise cell-cell interactions, have been investigated. Performance and algorithmic design choices of cell interactions in continuous and discrete space, where agents/entities are competing to interact with one another in a parallel environment are discussed, highlighting advantages and disadvantages of each implementation.

## Conclusion

We are encouraged by the growing support that the CMISF workshop is obtaining, and we believe that such support will become more and more stronger, as the use of computational methods and approaches to be used in the future in Silico Trials will become a common practice in the developing of new drugs. EMA and FDA have in fact expressed interest in the use of computational tools for the qualification of new drugs, and they are now moving towards the definition of the standards and rules to include computational models inside the qualification pipeline. To ensure that such themes are covered, we also included novel methods and approaches for in Silico Trials as a prominent topic of interest of the upcoming 3rd CMISF workshop that will be held soon in San Diego.

## Data Availability

Not applicable
